# Commentary: Grand challenge: ELSI in a changing global environment

**DOI:** 10.3389/fgene.2017.00135

**Published:** 2017-09-26

**Authors:** Ignacio Macpherson, Ignacio Segarra

**Affiliations:** ^1^Department of Humanities, International University of Catalonia, Barcelona, Spain; ^2^School of Pharmacy, University of Barcelona, Barcelona, Spain

**Keywords:** ELSI, human enhancement, genomic, bioethics, vulnerability, precautionary principle

In 2013, Greenbaum put forward the need to implement the Watson's ELSI initiative in 1988 upon the developing Genome Project (Greenbaum, 2013). The author reviewed the efforts made up to that moment and raised a number of possible essential improvements which would require a thorough revision to ensure the success of ELSI: (A) greater involvement of professionals not directly related to genomics; (B) greater efficiency to transmit ELSI initiatives through new technologies; (C) greater independence of ELSI science funding; (D) to internationalize ELSI development integrating other visions and cultures to face the problems; (E) the need to determine precise boundaries and red lines for any research progress taking into account society's outlook and (F) to extend the ELSI initiative to other fields of avant-garde science.

Four years later after their proposal, these ideas have materialized together with certain concerns. Perhaps because reflections of professionals from non-experimental sciences and disciplines (e.g., philosophers, humanists, legislators) as well as perspectives and points of view from different cultures have been taken into account to assess the implications. In addition, ELSI science began to permeate other research disciplines showing the imperative need for red lines. In this context, it seems to us that an additional problem has aroused which is no longer related to possible poor practical application, funding, extension or its inclusive openness to other cultures of ELSI projects. Rather, it seems intrinsic to its own concept and definition: the current scope of “ethical,” “legal,” and “social” terms.

Greenbaum's essay marks the starting point of this reflection including concern and alarm: the risk to be late. This was Oliver and McGuire's reflection in 2011 when he demanded a faster implementation of ELSI congresses resolutions upon the fast changes in genetics without knowing whether ELSI initiatives were being truly effective (Oliver and McGuire, 2011). The absence of an ELSI perspective in emerging research areas is also frequent (Garnett et al., 2011) likely influenced by the lack of method and the low effectiveness of literature reviews focused on diverse directions (Walker and Morrissey, 2014). Furthermore, a popular view of manipulation and fraud of scientific studies due to their apparent pursue of ideological (Nisbet and Markowitz, 2014) or even economic interests (Greenbaum, 2013) may arise. This milieu prevents a deep and balanced debate in the mass media: rather it generates information distrust and “media wars” to influence social opinion without their in-depth evaluation (McClain, 2017). Although social networks and communication channels are extremely fast, we think that the problem strives from the public's capacity to understand the significance of the ELSI dilemmas. This is evidenced when ethical issues combining genomics with neuroscience, “*neurogenomics*,” are raised: genomic editing, gene therapy, memory manipulation, genomic identity, online communities, etc. (Canli, 2015). All them seem to converge on a question that we think that is the big question that ELSI science must address: what is the purpose of it all? This was well evidenced in the Japanese Expert Panel on Bioethics when it assessed whether the creation of animal-human chimeras may alter the identity of human beings and therefore whether it should be allowed (Mizuno et al., 2015). Similarly, online sharing of individual genetic features raises social issues that affect everyone as a community. Genetic makeup may be exposed in social networks with unexpected consequences: will former racism based on phenotype surface once again through a new genotype discrimination? (Hoppe, 2013). Underlying these dilemmas we may see the apprehension upon the unknown because we do not know what we are changing. This is why it is imperative for the ELSI project to have a universal scope: local legislation is of limited use if it is permissive in another part of the globe, given the globalism of science.

The height of ELSI science stands out when the concept of Human Enhancement appears, whether cognitive (neuroenhancement), moral (moral enhancement), physiological (physical enhancement), genetic (genomic enhancement) or even species enhancement (transhumanism). This is illustrated with the formulation of a right to genetic improvement, is there a right not to improve? (Tamir, 2016). From here the subsequent question follows: is there a right to be perfect? A question which has been widely debated in the context of moral enhancement (Harris and Savulescu, 2015). The answer may be none, but with imprecise and vague reasons: we are aware that no one can force us to be better, but we do not know why (Tonkens, 2015). There seem to exist a conceptual vacuum that causes reasoning to be stagnant, unable to define well what perfection, autonomy or identity are. At this point, O'Connor and Nagel's (2017) emphasis or a broad concept of neuro-improvement is relevant, since any improvement initially aimed at the individual also affects society. This advocates that sometimes the solution may be relational, owing to the fact that human beings are 100% individual and social. If the driving force of human enhancement is individual interest (or individualism) it will begin to degrade and may end up perverting such initiative, due to lack holistic understanding of the relational dynamics. As Cabrera (2017) points out, human enhancement seems to be “very much about values, ideology, and political will.” We think that until that effort, including the purpose of the actions, is made the ELSI initiative is at risk of becoming a chimera.

Last, but not least, it is necessary to reflect on the precautionary principle (Gonzalvo-Cirac et al., 2013) and its social exemplification in research settings involving society, which in itself calls for rational, cognitive and in-depth reflection (Hildt, 2016). The problem then, becomes no longer genetic or neurological only, but moral: what do we mean as good for a human being? What is human perfection? What does it make individuals more human, regardless of being more or less intelligent, resilient or skillful? What makes an individual worthy of respect by others? How is enhancement regulated? Is science for societal good or the individual good prevails? There seem many questions which are addressed by those four words: Ethical (good), Legal (standards), Social (others), Implications (consequences) also known as ELSI science.

## Author contributions

IM and IS contributed to the discussion and writing of the manuscript.

### Conflict of interest statement

The authors declare that the research was conducted in the absence of any commercial or financial relationships that could be construed as a potential conflict of interest.
